# Pancreatic Cancer and Obesity: Molecular Mechanisms of Cell Transformation and Chemoresistance

**DOI:** 10.3390/ijms19113331

**Published:** 2018-10-25

**Authors:** Priscilla Cascetta, Alessandro Cavaliere, Geny Piro, Lorena Torroni, Raffaela Santoro, Giampaolo Tortora, Davide Melisi, Carmine Carbone

**Affiliations:** 1 Medical Oncology Unit, Azienda Ospedaliera Universitaria Integrata, 37134 Verona, Italy; priscillacascetta@gmail.com (P.C.); alessandro.cav@hotmail.com (A.C.); giampaolo.tortora@univr.it (G.T.); davide.melisi@univr.it (D.M.); 2 Digestive Molecular Clinical Oncology Research Unit, Section of Medical Oncology, Department of Medicine, University of Verona, 37134 Verona, Italy; lorena.torroni@univr.it (L.T.); raffaela.santoro@univr.it (R.S.); 3 Medical Oncology, Fondazione Policlinico Universitario A. Gemelli IRCCS, 00168 Roma, Italy

**Keywords:** obesity, pancreatic cancer, chemoresistance, inflammation

## Abstract

Cancer and obesity are the two major epidemics of the 21st century. Pancreatic ductal adenocarcinoma (PDAC) is one of the leading causes of death, with a five-year overall survival rate of only 8%. Its incidence and mortality have increased in recent years, and this cancer type is expected to be among the top five leading causes of cancer-related death by 2030 in the United States (US). In the last three decades, the prevalence of overweight people has boosted with a consequent increase in obesity-related diseases. Considerable epidemiologic evidence correlates overweight and obese conditions to an increased risk of several types of cancer, including PDAC. Besides being a risk factor for multiple metabolic disorders, the tumor-promoting effects of obesity occur at the local level via inflammatory mediators that are associated with adipose inflammation and metabolic or hormones mediators and microbiota dysbiosis. Although an excess of body mass index (BMI) represents the second most modifiable risk factor for PDAC with an increased cancer related-death of more than 20–40%, still little is known about the molecular mechanisms that underlie this strong association. In this review, we focused on the role of obesity as a preventable risk factor of PDAC, discussing the molecular mechanisms linking obesity to cancer initiation and progression. Moreover, we highlighted the role of obesity in defining chemoresistance, showing how a high BMI can actually reduce response to chemotherapy.

## 1. Introduction

### 1.1. Obesity and Pancreatic Cancer

Body mass index (BMI) is an easily measurable biometric parameter that is obtained as the ratio between weight, expressed in kilograms, and height, expressed in square meters. According to the criteria of the World Health Organization, a BMI <18.5 kg/m^2^ is defined as underweight, between 18–25 kg/m^2^ is defined as normal weight, between 25–30 kg/m is defined as overweight, and >30 kg/m^2^ is defined as obesity. Currently, obesity is one of the leading medical and socio-economic problems worldwide. 

In 2015, 107.7 million children and 603.7 million adults were obese worldwide. In the last three decades, the prevalence of obese and overweight people has boosted, with a subsequent increase of obesity-related diseases. The peak in the prevalence of obesity is recorded between 60–64 years of age in women and between 50–54 years of age in men [1]. Overweight and obesity are the main risk factors for many of the most frequent chronic diseases, including cardiovascular diseases, diabetes, chronic kidney disease, and osteoarticular diseases. Moreover, considerable epidemiologic evidence correlates excess body mass to increased risks of several types of cancer. In 2015, cancer represented 5.4% of all BMI-related deaths and 4.2% of BMI-related disability-adjusted life years [1].

PDAC is the fourth leading cause of cancer-related death in the United States (US), with an overall five-year survival rate of 8%. In the United States, 55,440 new diagnoses of PDAC with 44,330 deaths are expected in 2018 [2]. PDAC incidence and mortality have increased over the last years; so far, this cancer type is expected to be the second-leading cause of cancer-related death by 2030 [3].

PDAC risk factors can be divided into modifiable and non-modifiable risk factors. The non-modifiable risk factors include age, diabetes mellitus, genetic syndromes, and non-O blood group. Modifiable risk factors comprise smoking, obesity, dietary habits, inactivity, and misuse of alcohol [4]. 

Giving the decline of cigarette smoking, which represents the main PDAC risk factor, a possible explanation of the registered augmented incidence and mortality of PDAC may reside in an increased prevalence of obesity in the general population, especially in adulthood and childhood. Yuan et al [5] demonstrated that pre-diagnostic high BMI is associated with both increased risk of developing PDAC and mortality. Interestingly, they showed a statistically significant positive association between the pre-diagnostic BMI and stages of PDAC, demonstrating that obese patients present more frequently at the diagnosis with metastatic disease when compared with normal BMI [5].

To date, few studies have assessed the correlation between an excess of fat accumulation in the abdominal area (central obesity) and an increased risk of pancreatic carcinoma mortality [5,6]. In particular, in a pooled analysis of wide-cohort studies (20 cohorts, *n* = 1,564,218 participants), it was observed that waist circumference and waist-to-hip ratio, which are the most common anthropometric parameters for the estimation of central obesity, were associated with increased PDAC mortality regardless of BMI. Moreover, the association with PDAC mortality was higher when obesity was gained during early adulthood (ages 18–21 years). After early adulthood, only a considerable enhancement in BMI (>10 kg/m^2^) increased the risk of death for pancreatic carcinoma. In this analysis, an association between waist circumference and mortality emerges also in patients with normal BMI, suggesting an important role of visceral fat [7].

In another study, by measuring visceral obesity by computed tomography (CT) scan at the time of diagnosis, the authors demonstrated that visceral obesity was associated with worse OS (overall survival) and PFS (progression-free survival) as well as increased regional lymph nodes metastasis [8]. 

In a recent retrospective study in a large cohort of patients with severe obesity (average BMI >40 kg/m^2^), it has been shown that among obesity-associated tumors, the risk of PDAC (Hazard Ratio 0.46, 95% Confidence Interval 0.22–0.97, *p* = 0.04) was significantly lower in patients undergoing bariatric surgery compared with control patients [9]. In a genetically engineered mice model (GEMM) of pancreatic cancer (KC: “LSL-KrasG12D and PDX-1-Cre”), the increase in visceral fat, including intrapancreatic and peripancreatic fat, causes a systemic and local inflammatory state, with a greater production of pro-inflammatory cytokines, which are able to recruit immune cells in the pancreas and determine an acceleration of the tumor growth and a more aggressive tumor phenotype [10]. The main relevance of this study is the observation that in this GEMM of pancreatic cancer, high-fat-diet (HFD) and calories are associated with a higher inflammation rate in the visceral adipose tissue in the peripancreatic region, which is histologically and functionally different than the adipose tissue in other visceral regions.

Furthermore, the increase in visceral fat is associated with insulin resistance, which seems to have a relevance in PDAC development [11].

Obesity is also associated with increased operative risk in abdominal surgery. Data suggest that obesity is a risk factor for surgical complications after pancreaticduodenectomy (conventional Whipple’s procedure or the pylorus-preserving procedure). Obese patients who underwent pancreaticduodenectomy, both for malignant and benign pathology, often require a longer duration of surgery with a higher incidence of a pancreatic fistula and intraoperative bleeding [12]. The long-term oncologic outcomes of pancreaticduodenectomy in patients with a high BMI is unclear, with conflicting results within the few available reports [13,14].

Overall, these studies highlighted the role of obesity, especially visceral adiposity, as a risk factor for the development of PDAC and its mortality. In particular, the association between early adulthood obesity and higher PDAC incidence suggest a potential role of adipose tissue in the initial phases of tumor development. Therefore, educational campaigns on healthy lifestyles promoting the control of weight and anthropometric parameters are urgently needed for early prevention.

### 1.2. Cancerogens in Food: How Dietary Habits Are Related to PDAC

Dietary habits represent a risk factor for the development of PDAC. Studies have already reported a statistically significant positive association between red-meat consumption in men and PDAC, and this evidence seems to rely especially on red, grilled, or processed meat [15,16,17,18]. Many molecular mechanisms have been proposed to explain this evidence. For instance, processed meat often contains some preservative substances such as nitroso-based compounds (especially *N*-nitrosamines), which act as potent mutagens by alkylating the DNA and whose exposure has been correlated to the development of PDAC in mice [19,20]. Moreover, studies highlighted that cooking meat at high temperatures as utilized for grilled and barbecued meat might release a higher percentage of some carcinogenic substances such as heterocyclic amines (HCAs) and polycyclic aromatic hydrocarbons (PAHs) [16,17,19], which are capable of interacting with and damaging DNA, thus having a pro-carcinogenic role in the development of PDAC [21,22].

Even though all of these nutrients have been related to PDAC, an important risk factor in determining both obesity and PDAC is represented by a high-fat diet (HFD) [23]. Several HFD-related molecular mechanisms of pancreatic cell transformation have been reported. In 2013, Philip et al. [24] demonstrated that obesity-induced HFD acts as an environmental stimulus at the very early stages of carcinogenesis and contributes to transforming precancerous pancreatic lesions into more aggressive PDAC. In this study, mice expressing mutant (G12D) KRAS (LSL-Kras/Ela-CreERT mice) fed with a HFD showed a significantly higher rate of pancreatic intraepithelial neoplasia (PanIN) lesions, pancreatic inflammation, and fibrosis than the same mice fed a controlled diet, thus underlying that Kras activation might be necessary but not sufficient in transforming pancreatic cells. Moreover, mice expressing oncogenic Kras and fed a HFD had significantly higher levels of activated Kras and downstream phospho-ERK signaling, highlighting that a possible mechanism of action of HFD might reside in implementing Kras activation [24]. According to a recent study, the pancreatic cancer cells of HFD-induced obese mice expressing Kras also presented a dysregulated autophagy as well as some unique genetic alterations compared to controlled-diet mice, highlighting the possibility that under particular genetic circumstances, a HFD may play a role in controlling autophagy as well as determining genetic alterations [25].

Moreover, HFD-induced obesity seems not to affect the invasiveness of transformed pancreatic cells. Stark et al. [26] demonstrated that the expression of E-cadherin and consequently epithelial–mesenchymal transition (EMT) did not significantly differ in Kras-expressing mouse models when fed a HFD compared to a controlled diet, although a higher percentage of PanIN lesions and PDAC was seen in the first subgroup [26]. Conversely, recent studies showed an enhanced EMT in pancreatic cancer cells mediated by pro-inflammatory cytokines that were secreted in response to obesity; thus, further studies are needed to unequivocally demonstrate the correlation between obesity and PDAC invasive behavior. 

Furthermore, HFD may also act in a pro-tumorigenic way by inducing genetic alterations in pancreatic transformed cells. By the exome sequencing of PanIN lesions, Chang et al. [25] demonstrated that the DNA obtained from mice expressing Kras and fed a HFD were enriched with genetic variants that could not have otherwise been found in Kras mice receiving a controlled diet. These 4856 genetic unique variants (in 2986 genes) mainly affected some key pathways that were commonly implied in PDAC progression, such as transmembrane transport, PI3K-Akt signaling, insulin signaling, extracellular matrix-receptor interaction, solute carrier-mediated transmembrane transport, and G protein signaling pathways [25]. However, other studies are needed in order to elucidate the functional implication of these variants.

Moreover, a HFD may contribute to cell transformation by impairing DNA repair mechanisms. A recent study demonstrated that a HFD could enhance the DNA damage in transgenic mice overexpressing satellite-RNA sequences, probably by modulating DNA repair mechanisms. Initially, the authors demonstrated that the overexpression of mouse major satellite RNA (MajSATRNA) increases the number of genomic and mitochondrial DNA mutations in mouse primary cell lines derived from Kras-mutated PanIN tumors, leading to the cellular transformation of precancerous cells. Then, the same authors demonstrated that the DNA damage induced by both caerulein treatment and HFD impaired the nuclear localization of Y-Box Binding Protein 1 (YBX1), which is a component of the DNA damage repair machinery. Strikingly, this event also occurred in mice without significant weight gain [27]. Accordingly, the evidence in the literature has shown that in a wide subset of cancer types, HFD may also have a tumor-promoting role per se, independent from the association with obesity. Indeed, using in vivo models of obesity-resistant BALB/c mice orthotopically implanted with 4T1 mammary carcinoma cells, Kim et al. [28] demonstrated that tumor growth and progression was higher in those receiving a HFD compared to those receiving a controlled diet, with a higher macrophage infiltration into adipose tissue as well as serum levels of pro-inflammatory cytokines [28]. Furthermore, Soto-Pantoja et al. [29] demonstrated that a high-fat diet could promote tumor progression and tumorigenesis in GEMM models of colon cancer (APDACMin+) via a systemic and local pro-inflammatory effect, and that this result is present even in a short dietary exposure not determining obesity. In this study, the authors also highlighted that this result was obtained with a specific high-fat diet that was enriched in saturated and omega-6 polyunsaturated fatty acids [29]. Even though these studies were performed in tumor types different from PDAC, they highlighted the possibility that a HFD could represent per se a pro-tumorigenic status. 

HFD also acts by diminishing the defensive mechanisms that are normally present in the epithelial layer. Indeed, at a very early stage of tumorigenesis, single transformed cells compete with normal cells in the epithelial layer, and in most cases, they are rapidly removed from the epithelial layer through a mechanism called “apical extrusion”. When this mechanism fails, transformed cells may proliferate, generating cancer [30,31].

More recently, Sasaki et al. [32] generated a GEMM with Ras-V12 under TET-inducible epithelial specific promoter recapitulating a cell competition model in various tissues, such as the pancreas, small intestine, and lung. In this model, more than 65–90% of RasV12-expressing cells underwent apical extrusion from the small intestine, pancreas, and lung epithelia. Then, the authors analyzed the effect of a HFD on apical extrusion. Mice were thereby fed either a controlled diet or three months of a HFD, resulting in a significant decrease in the apical extrusion of Ras-V12 transformed cells in the pancreas and small intestine layer, but not in the lung, for the HFD subgroup. As expected, the maintenance of Ras-V12 transformed cells, resulting in a more frequent tumor development into the apical lumen of the pancreas and small intestine epithelium. Moreover, the authors obtained the same results; even with short-term exposure to a HFD (four days versus three months), they were not able to determine a significant increase in mice body weight, thus underlying that a HFD may have per se a tumor-promoting role, independent from obesity, as mentioned above. In order to understand the mechanisms through which a HFD could decrease apical extrusion, they further analyzed the mitochondrial membrane potential of Ras-V12 transformed cells, highlighting that a HFD could restore mitochondrial membrane potential in Ras-V12 transformed cells with a subsequent inhibition of their eradication from the epithelium.

It is widely known that different fatty acids may have an impact on tumor growth. For mammals, essential fatty acids are mainly represented by omega-3 and omega-6 fatty acids, which can be both oxidated to acetyl-CoA. Nonetheless, omega-6 fatty acids may also be transformed into prostaglandins and leukotrienes, thus having an important pro-inflammatory role and a subsequent pro-tumor activity. Indeed, omega-3 fatty acids may be metabolized into eicosapentaenoic acid, which is demonstrated to have an anti-inflammatory effect both in vivo and in vitro. As a consequence, tumor promotion may be especially enhanced by the assumption of HFD enriched with omega-6 fatty acids rather than with the omega-3 ones [32].

In their work, Sasaki et al. [32] also showed that the reduction of apical extrusion was more evident when mice were fed an omega-6 fat diet such as soybean oil, compared to an omega-3 fat diet such as linseed oil. More importantly, in this study, data on higher inflammatory cytokines as well as macrophage infiltration in the first subgroup of mice, together with the evidence of an increased frequency of apical extrusion in HFD mice treated with aspirin, demonstrated a link between HFD and inflammation in pancreatic cancer. Indeed, inflammatory cytokines as well as macrophage infiltration was higher in the first subgroup of mice, thus demonstrating a link between HFD and inflammation in pancreatic cancer, so far that aspirin treatment increased the frequency of apical extrusion in HFD mice [32]. 

Overall, different mechanisms may explain the correlation between obesity, HFD, and the development of PDAC. In pre-clinical models, obesity-induced HFD seems to play an important role by enhancing inflammation and Kras activation rather than increasing invasiveness. Moreover, HFD may also act by altering DNA. Accordingly, the presence of specific genetic variants in PanIN lesions obtained from mice previously fed with HFD has been demonstrated. Finally, HFD may diminish normal defensive mechanisms inside the epithelial layer, such as apical extrusion. Interestingly, data also evidenced that HFD may represent per se a pro-cancerous agent in PDAC cells, independent from obesity, as apical extrusion was lower even after a short-term exposure to HFD (four days). Different fatty acids may form the diet, and their difference may have an impact on tumor growth, as metabolites obtained from omega-6 fatty acids have a pro-inflammatory function that is typically absent in metabolites obtained from omega-3 fatty acids. Differences in the composition of HFD (omega-3 versus omega-6) may also play a central role in pro-tumor activity. 

## 2. Obesity and Inflammation

PDAC is characterized by a huge component of desmoplastic fibrotic reaction due to extensive extracellular matrix (ECM) deposition by activated pancreatic stellate cells (PSC) [33]. The fibrous component causes compression on the blood vessels with relative vasal collapse and consequent heterogeneous tumor hypoperfusion. The presence of tumor hypoperfusion, due to the desmoplastic reaction, constitutes a barrier to the delivery and efficacy of antitumor agents [34].

Obesity is associated with an increase in the adipose content of the normal pancreas (pancreatic steatosis), which determines an inflammatory microenvironment by increasing the production of cytokines, the extracellular matrix, and fibrosis. In obese patients, the intrapancreatic fatty infiltration is correlated with the development of PanIN and PDAC [35,36]. The intrapancreatic fatty infiltration is the main histopathological feature of the hereditary pancreatitis, secondary to germline mutation in *PRSS1* gene, which is an autosomal dominant disease that is associated with a considerably increased risk of developing PDAC. In these patients, the intrapancreatic fatty infiltration appears to be caused by inflammatory processes secondary to intercurrent episodes of acute pancreatitis [37]. Adipocytes are endocrine cells that produce many molecules, including hormones, growth factors, and cytokines [38].

In general, in obese patients, adipose tissue is characterized by hypertrophy, hyperplasia, and an increase in the number of preadipocytes [39,40]. In physiological conditions, preadipocytes produce inflammatory cytokines and chemokines that determine a pro-inflammatory environment with the attraction and activation of macrophages and endothelial precursors. The main pro-inflammatory cytokines are leptin, TNFα, RBP-4, VEGF, ANGPTL2, IL-6, IL-8, IL17, CCL2, and CCL5 [41,42]. On the other hand, the infiltrated macrophages produce IL-6, IL-8, CCL2, and CCL5 [41,42,43]. The inflammatory environment supports the proliferation of preadipocytes and the formation of blood vessels [44]. In obese patients, adipose tissue leads to chronic inflammation through the activation of innate immunity. This chronic inflammation triggers preadipocytes’ proliferation, impairing their differentiation [45]. The two main cytokines produced by adipose tissue are adiponectin and leptin. Whereas adiponectin has an antiproliferative and anti-inflammatory function, leptin has a mitogenic and inflammatory function. Furthermore, leptin is also involved in the differentiation of monocytes in macrophages. The inflammatory environment caused by the adipose dysfunction associated with obesity represents a general systemic situation that also occurs at the local level. This inflammatory state in the pancreatic microenvironment is associated with the fibroinflammatory desmoplastic reaction, epithelial mesenchymal transition, immunosuppression, chemoresistance, and tumor progression [40,46].

Adiponectin is implicated in the maturation of preadipocytes to adipocytes [47]. While the mature adipocyte produces both leptin and adiponectin, the preadipocytes produce only high levels of leptin [48]. In obese patients, high levels of free fatty acids (FFA) are able to activate preadipocytes and inflammatory cells by inducing toll-like-receptor 4 (TLR4) signaling [49]. Elevated leptin expression levels induce in PDAC cells the expression of Notch receptors and ligands (Notch1, DLL4, JAG1, survivin, and HEY2) and PDAC stem cell markers (CD24/CD44/ESA, ALDH, CD133, Oct-4). Interestingly, PDAC cell lines and human PDAC tissues express the leptin receptor Ob-Rb. Treatment with leptin enhance the migration and invasion of PDAC cells as well as their metastatic potential in in vivo PDAC models upregulating matrix metalloproteinase-13 (MMP-13) via the JAK2/STAT3 signaling pathway [50]. It has been reported that the cross-talk between Notch and leptin appears to be mediated by IL-1. The activation of Notch’s non-canonical and canonical pathway is correlated with transformation, proliferation, tumor progression, epithelial to mesenchymal transition (EMT), and chemoresistance [51]. Although the study was performed in a pre-clinical model of breast cancer, the authors conclude that this mechanism could be common to different cancers, including pancreatic cancer. Moreover, in obese patients, the cytokines produced by adipocytes and immune cells could promote the survival of cancer stem cells (CSCs) through the activation of the Notch3 and IL-6/JAK2/STAT3 pathways, as demonstrated in other cancer models [52,53]. It has been demonstrated that the same cytokines storm in the tumor microenvironment determines the constitutive activation of NF-κB pathways, which are implicated in inflammatory-driven EMT intensification [54], tumor proliferation, and cancer resistance [55,56]. With regard to fibrosis in PDAC, adipocytes have a high receptor density for angiotensin II type 1 receptor (AT1R), whose activation in a pro-inflammatory environment is associated with the pro-fibrotic pathway [57,58]. Angiotensin II type 1 receptor (AT1R) is a major player in the signal transduction of the renin-angiotensin system, and the activation of this signaling contributes to the progression of visceral obesity. The AT1R-associated protein (ATRAP) promotes AT1R internalization from the cell surface into the cytoplasm, avoiding the overactivation of tissue AT1R signaling and preventing the development of diet-induced visceral obesity and fibrosis [59]. In addition, the fatty infiltration observed in obese patients participates in the vasal compress with a decrease in blood flow and consequently hypoxia [60]. In two genetically engineered mice models of PDAC (KPC: “Ptf1-Cre/KrasLSL-G12D/+/p53LSL-R172H/+” and iKRAS: “Ptf1-Cre/ROSA26-LSL-rtTa-IRES-eGFP/TetO-KrasTetO-LSL-G12D/p53L/+”), Incio et al. [40] demonstrated that cancer-associated adipocytes (CAAs) through the production of pro-inflammatory cytokines, mainly IL1B, determine the activation of PSC, the recruitment/activation of tumor-associated neutrophils (TANs) with decreased CD8^+^ T cells, and increased Treg with a relative immunosuppressive microenvironment that promotes, in turn, tumor growth and metastasis. IL1B was also produced by TAN and PSC by generating a cross-talk that amplified and retains fibroinflammatory processes [40,61]. 

They also found that obesity is associated with an increase in the pathway of AT1, and, in obese animals, its inhibition with losartan or with the genetic block has an antitumor effect and aids the efficacy of chemotherapy with 5FU. AT1 is expressed both on adipocytes and on PSC. Turning off the signaling pathway of AT1 decreased the production of IL1B that is produced both by adipocytes and PSCs. Moreover, in obese murine models, the inhibition of AT1 determined the reduction of hypoxia and EMT markers, supporting the hypothesis that the pathway of AT1 is associated with the development of desmoplasia. Supplementary analysis of tumor samples from more than 300 PDAC patients revealed enhanced desmoplasia alone in samples from obese patients. The excess body weight was correlated with reduced responses to chemotherapy [40].

Lipocalin-2 (LCN2), which is secreted by adipocytes, neutrophils, macrophages, and tumor cells, is an adipokine expressed in the serum of obese and PDAC patients [62].

Gomez-Chou et al. [46] demonstrated in PDAC the existence of a link between obesity and inflammation in the tumor microenvironment through the expression of LCN2. Indeed, cells within the adipose tissue contributed to the secretion of pro-inflammatory factors, such as LCN2, which could promote PDAC development [46].

A high expression of circulating LCN2 was observed in the serum after HFD consumption in a GEMM (KPC: “PDX-1-CRE, LSL-KRasG12D, LSL-Trp53−/−”) of PDAC, and it has been demonstrated that this cytokine could activate fibroblast into PSCs [46]. 

Although PSCs do not synthesize LCN2, stromal cells expressed significantly higher levels of the LCN2-specific receptor SLC22A17 compared to PDAC cell lines and normal pancreatic epithelial cells. LCN2 induced PSCs to secrete higher levels of the pro-inflammatory molecules complement component 5a (C5a), IL-6, stromal cell-derived factor 1 (SDF-1), interleukin-8 (IL-8), Intercellular Adhesion Molecul 1 (ICAM-1), IL-1β, MMP-1, MMP-3, and MMP-9, and alpha-smooth muscle actin (α-SMA) [46,62,63,64].

In addition, in a Kras-mutated murine system, they observed an increase of macrophages in the animals expressing *Lcn2* compared to those with deleted *Lcn2*. It has been demonstrated that at an early stage of pancreatic carcinogenesis, tumor-associated macrophages (TAMs) infiltrate the PDAC stroma, suggesting that the presence of TAMs is a pivotal and crucial initial process in PDAC development. Gomez-Chou et al. [46] did not demonstrate any correlation between BMI and plasma levels of LCN2 in patients with metastatic PDAC. However, PDAC patients at the time of diagnosis had advanced disease with substantial weight; furthermore, LCN2 could play a major role in tumor progression in the early stages compared to the advanced stages. According to the authors, these are the main reasons for the absence of the association between BMI and plasma levels of LCN2 [46].

Several studies suggest that adipocytes in an inflamed tumor microenvironment may dedifferentiate into fibroblast-like cells that can promote extracellular matrix remodeling, tumor growth, and tumor progression [60,65,66,67]. WNT5a, a non-canonical signaling pathway of WNT, plays a key role in the dedifferentiation of adipocytes [65]. The upregulation of WNT5a participates in the formation of a micro-inflammatory environment. On the other hand, adipocytes in a pro-inflammatory environment produce IL-6, which increases the expression of WNT5a through the STAT3 signaling pathway [68]. Recently, we demonstrated that in models of pancreatic preneoplastic lesions, adipocytes could induce EMT and aggressiveness by orchestrating a complex paracrine signaling of soluble modulators of the non-canonical WNT signaling pathway that determines, in turn, the activation and nuclear translocation of ROR2 (receptor tyrosine kinase-like orphan receptor 2). ROR2 is a Frizzled family protein that can bind to several WNT ligands and activate both the canonical and non-canonical WNT signaling pathways [69]. Interestingly, both the mRNA and protein expression of ROR2 was significantly higher in PDAC than in normal pancreatic tissues, and strong evidence revealed that ROR2 expression in tumor cytoplasm or stromal cells was significantly associated with malignant features and reduced survival in PDAC and strongly correlated with the poor prognosis of PDAC patients [70]. 

Overall, in PDAC, cancer-associated adipocytes interacting with the cells of innate immunity through inflammatory cytokines, including IL-1b and LCN2, determine an immunosuppressive environment, due to the increase in TANs and Treg, and a decrease in CD8^+^ lymphocytes and a fibrous environment that participates in tumor progression and intrinsic chemoresistance. This dialogue between inflammation, related obesity, and cancer is self-maintaining and self-amplified. In fact, the adipocytes in an inflammatory environment, which the obese patients seem to have most often, are able to dedifferentiate in fibroblast-like cells and implement the desmoplastic component with relatively few obstacles to the arrival in the tumor bed of chemotherapeutic drugs. The increase in leptin instead in obese patients with PDAC increases the expression of matrix metalloproteases, especially MMP-13, with the acquisition of a more invasive tumor phenotype.

## 3. Microbiota, Obesity, and Cancer

The human microbiome refers to all of the bacteria, archaea, fungi, protists, and viruses that inhabit our body. It has been demonstrated that a human body consists of 3.9 × 10^13^ microorganisms that share our body as commensal, symbiotic, and pathogenic microorganisms, and this wide variety of microorganisms is guaranteed thanks to the immune tolerance [71].

Yet, their presence is essential to us, playing an important role in metabolizing glycans, amino acids, and xenobiotics, and in synthesizing vitamins and other nutrients [72]. The composition of human microbiota, and especially gut microbiota, is constantly influenced over time by other factors such as aging, health status, and dietary habits [73], and recent studies have demonstrated that a low diversity in the microbiome is correlated with some human diseases such as allergy, arthritis, inflammatory bowel diseases, and even neuropsychiatric disorders, perhaps as a consequence of altered immune systems. Microbiota may also have a role in controlling food intake, glucose, and lipid metabolism, thus playing an active part in determining obesity [74].

In this sense, recent studies have demonstrated that some microbial compounds such as short-chain fatty acids (SCFAs), which are typically considered to be produced from non-digestible carbohydrates, may in turn act both in the neighborhood and in distant host cells, representing a source of energy and determining a higher secretion of PYY, GLP1, and GLP2, which are hormones that are notably involved in regulating the sense of hunger [75].

Most of the microbiota resides in the gut. Studies have demonstrated a link between gut microbiota composition and BMI. In fact, the transplantation of gut microbiota obtained from obese mice into lean mice was sufficient to improve their BMI, as the obese microbiome was more capable of digesting nutrients that wouldn’t be otherwise absorbed by the host [76].

Evidence has also highlighted the role of gut microbiota in the development of cancer via different mechanisms of action, among which the production of lipopolysaccharide (LPS) seems to play a central role. In fact, under specific circumstances, the gut microbiota may release LPS and other pro-inflammatory substances in a circle which then reach their targeted tissue upon activating the NF-κB pathway, which is a common signaling pathway that is implied both in inflammation and cancerogenesis. Interestingly, it has been observed that obesity is associated with release of pro-inflammatory cytokines, such as IL-6, IL-1, and TNF, which turn activate the same NF-κB pathway downstream, thus highlighting the possibility that gut inflammation and obesity-derived inflammation may converge in a pro-carcinogenic signaling inside the cells [77].

Under normal circumstances, the release of LPS in a circle (a condition known as endotoxaemia) is somehow prevented thanks to some protective host substances, such as mucus, IgA, and antimicrobial peptides. On the contrary, the absence of this equilibrium (a condition known as “dybiosis”) would determine an augmented endotoxaemia, leading to an improved risk of developing cancer [78].

In this sense, it has been postulated that HFD and obesity may represent environmental causes of dysbiosis, thus leading to an augmented endotoxaemia. In fact, LPS produced by gut microbiota may overcome the epithelial layer toward targeted tissues via chylomicrons; therefore, HFD may indirectly enhance metabolic endotoxaemia. Moreover, it has been demonstrated that the composition of gut microbiota differs in obese and lean people; obese people are enriched in *Firmicutes* when compared to lean ones with an augmented mucous layer thickness directly correlated with BMI [79,80,81].

Although obtained from cancer types that are different from PDAC, these data highlight a possible correlation between obesity, the gut microbiome, and cancer development.

Evidence regarding the gut microbiome and PDAC are very few, so no strong conclusion could be obtained. 

Recently, Ren et al. [82] demonstrated that the gut microbiota could distinguish PDAC patients from healthy people, with an increase of bacteria LPS producers in the first group. To further support a possible link between metabolic endotoxaemia and PDAC, studies have demonstrated that blocking the LPS receptor TRL4 resulted in a diminished development of PDAC [82]. 

Apart from gut microbiota, so few studies have highlighted a possible relation between PDAC and periodontal disease, that little known about whether and why there would effectively be a correlation between periodontal disease and the risk of PDAC [83,84]. In a prospective cohort study, Michaud et al. [83] examined the levels of antibodies against oral bacteria in 405 cases of patients who further developed PDAC and compared the same levels of antibodies to 416 controls, finding that people with higher level of antibodies against *Porphyromonas gingivalis* had up to a twofold higher risk of developing PDAC compared to people with low levels of these antibodies [83]. Nonetheless, other pathogens might be implied in augmenting the risk of PDAC. Comparisons between healthy and PDAC cases have also identified two oral bacteria (*Neisseria elongata* and *Streptococcus mitis*), whose concentration in saliva seems to be higher in PDAC patients compared to healthy patients [84]. However, a subsequent study did not confirm these data, but rather found a higher concentration of *Leptotrichia* spp. and *Porphyromonas* spp. in PDAC cases [84].

Besides, it is still not clear how periodontal disease might be effectively correlated with the development of PDAC. Evidences suggest that the dissemination of pathogens as well as a diversity in oral fauna might alter systemic cytokines in a pro-inflammatory panel, thus being indirectly correlated to higher risk of PC, but this evidence is not yet completely clarified [85]. According to others, LPS released by oral pathogens may work on immune cells in the tumor microenvironment, rather than being released in a circle [84].

Less is known regarding how obesity would possibly correlate with both periodontal disease and PDAC. According to some hypotheses, the high levels of TNF that are commonly associated with obesity might lead to a hyper inflammatory state with a subsequent augmented risk of periodontal disease, although no strong evidence are available supporting this assumption [11,86].

Up to date, few evidence regarding the gut microbiome and PDAC exist, so that no solid conclusion could be obtained. Moreover, some limited studies indicated a slight correlation between periodontal diseases and PDAC, with no strong explanation regarding how and which could be a real link between obesity, PDAC, and the gut microbiome.

## 4. Hormones

Obesity and being overweight are associated with hyperinsulinism and insulin resistance. Hyperinsulinism is a known risk factor for PDAC [11]. Insulin, through the binding to its receptor or to the insulin-like growth factor receptor (IGFR), activates the mTOR, PI3K, and MAPK pathways [87], inhibits apoptosis, and stimulates tumor proliferation and angiogenesis [88,89].

IGF-1 and 2 are systemic growth factors that are produced primarily by the liver in response to growth hormone (GH) levels. It is interesting to note that there is no linear correlation between IGF and obesity. IGF is paradoxically greater in overweight people than in obese people. In fact, in high obesity, levels of IGF determine a negative feedback on the production of pituitary GH with a consequently lower hepatic production of IGF [90]. Recent evidence suggested that IGF binding to its target receptors plays an important role in pancreatic tumor fibrogenesis. In fact, in PDAC cells, Kras-mutated Sonic Hedgehog activation increases the expression of IGF-R1 on tumor cells and the secretion of IGF1 by stellate pancreatic cells [91,92]. 

The PDAC hypoxic tumor microenvironment also induced the production of IGF1 by cancer-associated fibroblasts (CAFs) and the expression of IGF1R in PDAC cells. In PDAC, it is reported that the paracrine interaction between CAFs and PDAC cells, which is mediated by IGF/IGFR1 signaling, is involved in tumor motility [93]. Furthermore, in PDAC, the IGF/IGFR axis stimulates stem-like mesenchymal cells to produce CCL5/CCR5, resulting in a further cross-talk between stroma and inflammation. The importance of this dialogue is shown by the high levels of TAMs, which are associated with high levels of IGF/IGFR expression and poor prognosis in PDAC [94,95]. 

The adipose tissue is able to produce estrogen. In postmenopausal women and men, adipose tissue is the main product of estrogen; indeed, in obese patients, there are high levels of endogenous estradiol and estrone [96]. In the bloodstream, the sex hormones are transported by the sex hormone binding globulin (SHBG), which is synthetized in the liver. Hyperinsulinemia, which is one of the characteristics of obese patients, leads to a reduction in hepatic SHBG synthesis [97]. The reduction of the SHBG involves an increase in the free share of sex hormones (both estrogen and testosterone) in the blood. Despite this greater amount of sex hormones in obese patients, estrogen seemed to inhibit pancreatic cancer cell lines proliferation [98], and the few available data do not suggest a significant association between exposure to sex hormones and PDAC [99].

In consideration of the current evidence, in PDAC, the IGF/IGFR1 signaling on stromal cells participates in the formation of a fibroinflammatory microenvironment and tumor motility. However, the function of sex hormones is still unclear, as well as their role in the molecular mechanisms of PDAC development.

## 5. Obesity and Chemoresistance

PDAC cells and the microenvironment are able to promote an important pro-fibrotic state, resulting in a complex stroma-desmoplastic reaction that is a key element of PDAC.

In this sense, it has been demonstrated that up to 90% of the total tumor volume is represented by the stromal compartment. Yet, it is still not clear which would be the role of this fibrotic reaction; some have highlighted the possibility that this could contribute to PDAC chemoresistance by diminishing the delivery of drugs in the PDAC microenvironment [100]. 

In this context, obesity may enhance this pro-fibrotic state via pro-inflammatory cytokines, resulting in a decreased permeability to dugs (see above). Moreover, it has been postulated that epithelial to mesenchymal transformation (EMT), which is a common feature for invasiveness, positively correlates with chemoresistance [54].

Studies have highlighted the possibility that leptin, which is a cytokine derived from adipose tissue and whose levels are 10 times higher in obese people, may enhance the expression of Notch receptors and ligands in many tumors, including PDAC [101].

Indeed, PDAC cells treated with 5FU showed a significantly higher proliferation rate and higher levels of EMT when contemporarily exposed to leptin, thus individuating a potential role of obesity in determining chemoresistance via EMT [51,54,102]. Moreover, leptin may also directly activate the NF-κB pathway, which is notably involved in tumor progression and chemoresistance [103]. Nonetheless, adipose tissue also produces adiponectin, which is another cytokine that is commonly known to have an antitumor activity. In vitro experiments have evidenced that adiponectin could inhibit EMT in NSCLC cell lines, thus having contrary effects compared to leptin [104]. Whether and which would be the final effect produced by these two opposites cytokines in obese people is still to be proven.

Apart from cytokines, the hormones that are secreted in response to food intake may have a role in controlling EMT and chemoresistance.

In vivo experiments have demonstrated that circulating levels of diet-induced IGF1 was correlated with tumor progression and EMT in a murine model of luminal breast cancer [105], and diet-induced obesity promoted tumor progression and EMT in murine models of breast cancer [106].

Yet, their role in determining PDAC and chemoresistance has not yet been clarified. As described above, studies have highlighted the role of HFD and obesity in altering normal microbiota, thus being a likely environmental cause of dysbiosis. Moreover, recent studies have demonstrated that the microbiota may influence the responsiveness to some oncological therapies, including immunotherapy and chemotherapy. Recently, Yu et al. [107] demonstrated an abundance of *Fusobacterium Nucleatum* in the colorectal cancer tissue of a patient with recurrence post chemotherapy and further validated *Fusobacterium Nucleatum* to be primarily involved in chemoresistance via the TLR4 and MYD88 pathways [107]. Interestingly, a higher amount of *Fusobacterium* spp. has also been found in the tissue of PDAC patients, and this bacterium has been correlated with a worst outcome, although other studies have not confirmed this data [108,109]. 

By examining 257 fecal samples from both control and obese individuals, Liu et al. [110] recently demonstrated that the fecal samples obtained from obese people had lower bacterial diversity than controls, with some differences in bacterial species between these two subgroups. Among these, *Fusobacterium* spp. was highly present in fecal samples obtained from obese people, whereas *Bacteroides* spp. was typically present in leaner people. Interestingly, this difference in gut microbiota composition decreased in people who subsequently lost weight, evidencing a dynamic system. Moreover, gavage with *Bacteroides thetaiotaomicron* protected mice against adiposity. Overall, these data support the hypothesis that any existing difference in gut microbiota between lean people and obese people may play a role in determining chemosensitivity [110].

However, more evidence is needed in order to further elucidate the mechanisms and correlation between obesity-induced dysbiosis and chemoresistance. Whatever would be the mechanisms of chemoresistance, obesity per se represents a condition of impaired responsiveness to chemotherapy. 

In this sense, authors have already highlighted that obesity determined detrimental results for the obtainment of a pathological complete response to neoadjuvant treatment in locally advanced breast cancer patients [111], and similar results were obtained for different tumor types in diverse settings of therapy [112,113,114]. This evidence could be the effect of several aspects, including diverse pharmacokinetic and pharmacodynamics in this subgroup of patients. 

Obese people are often under-treated, and a correct dosage of chemotherapy for this subgroup of patients is still a challenging issue, so far that specific American Society of Clinical Oncology (ASCO) guidelines are available for helping clinicians [115]. 

Moreover, fatty tissue may alter blood circulation and ventricle function, with subsequent changes in volume distribution, as well as renal and hepatic function [116,117]. As previously examined, the drug delivery might also be affected. In this context, leptin was also found to be positively correlated with cancer steam cells’ expansion and the expression of ABC protein transporters, which are other notable mechanisms of chemoresistance [118]. Given the known relationship between PDAC and obesity, further studies are necessary to better understand the dual relationship between obesity, PDAC, and chemoresistance. 

Overall, obesity represents per se a condition of impaired responsiveness to chemotherapy; different pharmacokinetic and pharmacodynamics are often implicated to explain this evidence. Pre-clinical studies have demonstrated a possible correlation between obesity-induced hormones (leptin and IGF-1) and PDAC chemoresistance via augmented epithelial-to-mesenchymal transformation (EMT). Obesity-derived dysbiosis with subsequent altered microbioma could eventually further explain this evidence, although other studies are needed.

## 6. Conclusions

Obesity is considered to be one of the most important risk factors for many types of cancer, including PDAC. Many cohort studies have shown a strong connection between obesity and pancreatic cancer incidence; however, the molecular mechanisms linking obesity to PDAC is still under extensive investigation. This is due both to the physiological and physiopathological role of adipose tissue as an endocrine organ and as potential reservoir of chronic inflammation, pro-inflammatory cytokines, growth factors, and cell metabolites. This complexity is reflected in the many possible ways in which the adipose tissue could influence pancreatic cell transformation, cancer progression, and resistance, which in turn lead to a dramatic increase in disease aggressiveness, poorer response to treatments, and a decrease in overall patient survival. 

In this review, we summarized the main molecular mechanisms that might underlie the association between excess weight and pancreatic cancer. Moreover, we highlighted the influence of nutrients and microbiota dysbiosis in PDAC. 

Therefore, further studies are needed in order to clarify the role of adipose tissue in carcinogenesis and the aggressiveness of pancreatic cancer. However, the control of becoming overweight or obese through lifestyle may be able to prevent pancreatic cell transformation and/or improve the outcome of PDAC patients.
